# Electronic Quality of Life Assessment Using Computer-Adaptive Testing

**DOI:** 10.2196/jmir.6053

**Published:** 2016-09-30

**Authors:** Chris Gibbons, Peter Bower, Karina Lovell, Jose Valderas, Suzanne Skevington

**Affiliations:** ^1^ Cambridge Centre for Health Services Research University of Cambridge Cambridge United Kingdom; ^2^ Manchester Centre for Health Psychology University of Manchester Manchester United Kingdom; ^3^ The Psychometrics Centre University of Cambridge Cambridge United Kingdom; ^4^ Centre for Primary Care University of Manchester Manchester United Kingdom; ^5^ School of Nursing, Midwifery and Social Work University of Manchester Manchester United Kingdom; ^6^ Collaboration for Academic Primary Care University of Exeter Exeter United Kingdom

## Abstract

**Background:**

Quality of life (QoL) questionnaires are desirable for clinical practice but can be time-consuming to administer and interpret, making their widespread adoption difficult.

**Objective:**

Our aim was to assess the performance of the World Health Organization Quality of Life (WHOQOL)-100 questionnaire as four item banks to facilitate adaptive testing using simulated computer adaptive tests (CATs) for physical, psychological, social, and environmental QoL.

**Methods:**

We used data from the UK WHOQOL-100 questionnaire (N=320) to calibrate item banks using item response theory, which included psychometric assessments of differential item functioning, local dependency, unidimensionality, and reliability. We simulated CATs to assess the number of items administered before prespecified levels of reliability was met.

**Results:**

The item banks (40 items) all displayed good model fit (P>.01) and were unidimensional (fewer than 5% of t tests significant), reliable (Person Separation Index>.70), and free from differential item functioning (no significant analysis of variance interaction) or local dependency (residual correlations < +.20). When matched for reliability, the item banks were between 45% and 75% shorter than paper-based WHOQOL measures. Across the four domains, a high standard of reliability (alpha>.90) could be gained with a median of 9 items.

**Conclusions:**

Using CAT, simulated assessments were as reliable as paper-based forms of the WHOQOL with a fraction of the number of items. These properties suggest that these item banks are suitable for computerized adaptive assessment. These item banks have the potential for international development using existing alternative language versions of the WHOQOL items.

## Introduction

Improving patient-centered care (PCC) is a key strategic priority for health care systems worldwide due to the increasing burden of non-communicable chronic disease and ageing populations [1]. In the United States, the Institute of Medicine enshrines PCC as one of the six elements of high-quality care [2]. In the United Kingdom, the National Health Service (NHS) Outcomes Framework provides a new focus on patient outcomes, rather than processes of care—a vision grounded in PCC and shared decision making [3,4]. Improving quality of life (QoL) and satisfaction with care for patients with chronic conditions is central to the NHS Outcomes Framework’s objectives [5].

Increasing priority placed on PCC reflects a longstanding movement towards patient-centered metrics and away from sole reliance on disease-centered measures of severity, impact, and burden [6]. Such patient-centered metrics include satisfaction [7], activation [8], and subjective QoL [9]. Subjective QoL is of special interest as it seeks to quantify “an individual’s perception of their position in life in the context of the culture and value systems in which they live and in relation to their goals, expectations, standards and concerns” [9].

Patient-reported outcome measures (PROMs) are measurements of any aspect of a patient’s health status that come directly from the patient, usually through a paper-based questionnaire scale [10,11]. Measures of subjective QoL provide a comprehensive assessment of the patient’s life encompassing physical, psychological, social, and environmental factors, which are rated as useful by clinicians [12]. These measures may alert clinicians to a patient’s concerns and prompt a discussion between the two parties about these issues [13,14].

Clinical trials assessing PROM feedback report improvements in identification of clinical issues, emotional well-being, patient-centered discussions, and symptom recognition in pediatric, oncology, and respiratory settings [15-19]. We are unaware of any published randomized controlled trials that have used the World Health Organization’s Quality of Life (WHOQOL) instruments to evaluate the impact of assessment and feedback on patient outcomes (search strategy published in [20]). A recent pilot study demonstrated modest benefits of WHOQOL feedback on psychological QoL alongside positive comments on the perceived usefulness of sharing this information with doctors [21,22]. These results indicate some promise for this developing field.

However, the overall level of evidence for the impact of using PROs in clinical practice is mixed [11,14]. In mental health research, the effectiveness of PROM interventions appears to be mediated by the quality of the feedback [23]. A Cochrane review is planned to assess the evidence relating to the use of the PROMs in clinical practice [20].

Despite the potential benefits of PROM administration, their use is frequently dismissed in many medical settings including family practice [24], which may be partially attributed to the impracticality of administering paper-based questionnaires in a time-pressured environment. Recent research has highlighted the time- and resource-consuming issues of data storage, dealing with missing data, and analyzing results as potential barriers to the uptake of paper-based PROMs [25]. In the research setting, the length of questionnaires is negatively associated with response rate [26], indicating a general preference for shorter assessments, which may be adversely affecting implementation in tandem with other human, financial, and logistical barriers. A lack of clear instruction, training, and feedback that is linked to specific clinical action may also contribute to poor implementation of PROMs in clinical practice [11].

The increased availability of modern computing technologies (eg, smartphones and tablets) provides an opportunity to computerize PROM administration, which has previously been primarily paper-based. In addition to practical advantages of not having to score and interpret paper-based questionnaires, administration using a computer adaptive testing (CAT) algorithm is a quicker and potentially more relevant and accurate method of assessing patient reported outcomes [27,28]. The development of open-source platforms for deploying cloud-based CATs both on the Internet and within apps [29,30] allow researchers and clinicians to create and deploy CATs and presents new opportunities to disrupt traditional forms of PRO assessment and feedback and to deliver them directly to patients at scale.

In the United States, the patient-reported outcomes assessment system (PROMIS) program has developed computer adaptive tests for fatigue, pain, depression, and health-related QoL among other things *,* which are rigorously validated but unfortunately are currently limited to PROMIS-validated questionnaires only, which do not include subjective QoL [31].

CAT relies on model parameters derived from psychometrically calibrated item banks. Item bank information, including item parameters, is obtained by fitting scale data to item response theory (IRT) models, of which the Rasch model is a special case. The Rasch model has been widely used to assist the development and assess the psychometric properties of QoL scales [32,33], item banks for computer adaptive tests [34,35], short-form questionnaires [36,37], and for developing clinically oriented content-based interpretation tools. The Rasch model is closely related to other IRT models, but it has stricter assumptions that can result in concise banks of items devoid of uninformative or unnecessary items and that may yield the highest standard of measurement, including specific objectivity [38-40]. IRT affords some advantages over “classical test” methodologies. Most significantly, IRT models allow PROMs to be accurately administered using any number or combination of items from the original scale. This allows CAT algorithms to select the most relevant and informative items for the test taker [41].

We hypothesize that the application of item response theory and computer adaptive testing algorithms to QoL scale data will improve precision (ie, reliability) and efficiency (ie, the number of items needed to be administered). These improvements will be driven by the removal of unnecessary items during the calibration of the item bank using IRT and the “intelligent” administration of items using CAT algorithms. The study used items from the WHOQOL’s 100-item measure to create four item banks to measure QoL in physical, psychological, social, and environmental domains and to test the performance of these item banks using simulated CAT.

## Methods

### Population

We conducted the current analysis on data collected from 320 people living in the United Kingdom [9]. The population consisted of 162 females (51%), 260 “sick” people (balanced across International Classification of Diseases-10 categories I-XVIII), and a mean age of 44 years (SD 17). Detailed descriptions of the sample may be found elsewhere [9]. English is the development language of the WHOQOL measures, which are all designed to be internationally relevant, but there is some evidence that differential item functioning (DIF) between different countries exists for many items within the WHOQOL-100 [32]. We, therefore, chose to create item banks that will be psychometrically accurate for use in the United Kingdom in the first instance.

### Measures

#### WHOQOL-100

The WHOQOL-100 is a generic 100-item measure of subjective QoL designed for use across a spectrum of populations, including sick and well people. The original scale is scored as 25 facets representing six domains of quality of life (physical, psychological, emotional, social, independence, and spiritual) [42,43]. Other versions of the WHOQOL, including the WHOQOL-BREF, include the same facets to represent four domains of QoL (physical, psychological, environmental, social) [6,44]. Four items in the WHOQOL-100 represent general QoL and overall health. High scores in each domain (recoded for negatively worded items) indicate a better QoL than lower scores in the same domain. Respondents judge their quality of life over the previous 2 weeks. The international WHOQOL-100, as well as the UK national instrument, show excellent psychometric qualities of internal consistency, reliability, and construct validity [9,42,43]. In our research, the domains and facets of the WHOQOL-100 were arranged to mirror the four-dimension structure of the popular WHOQOL-BREF measure [6]. This structure has been empirically supported using structural equation modeling [42] and will facilitate comparisons between the new item banks and the large body of research that has employed the shorter four-dimensional measure.

Previous studies have applied data from the WHOQOL instruments to IRT resulting in both unidimensional and multidimensional solutions [32,45,46]. These studies uncovered issues relating to category threshold ordering, DIF between countries, and item redundancy (see Multimedia Appendix 1).

### Analysis

#### Item Response Theory

We assessed the advanced psychometric criteria and estimated item bank parameters by fitting scale data to the Partial Credit Model (PCM), a polytomous extension of the Rasch model suitable for Likert-type data [47]. Scalability and monotonicity were assessed using Mokken analysis before the more rigorous tests of PCM assumptions. Both the Mokken and Rasch models can be seen as probabilistic extensions of the deterministic Guttman scaling model. The probabilistic version is better suited to psychological constructs and real-world data [48]. Mokken analysis is done prior to Rasch analysis to ensure that the scale structure is consistent with the Rasch model (ie, item response probabilities increase monotonically in line with the level of the underlying trait). The combination of the two methodologies in this order is recommended and has been shown to be useful in previous research conducted by members of our group [37,49]. Where scale data did not fit either the Mokken or the PCM, an iterative process of scale improvement was undertaken by removing items that violated the assumptions of either model. The iterative process involved stepwise assessments of scalability (indicated by a Loevinger’s Ho value >.3), category threshold ordering, item fit to the PCM (chi-square *P*>.010), fit residuals (fit residuals within ±2.5), local dependency (residual correlations <.10), and DIF (no significant analysis of variance interactions by demographic group). Items that violated any of the above assumptions were individually removed, and the remaining items were reanalyzed. Disordered thresholds were collapsed for adjacent categories, while ensuring anchor semantics remained logical (ie, “Agree” would not be collapsed into “Neither Agree nor Disagree”). This process was repeated until no items failed to meet the assumptions of the PCM: presented category disordering, misfit to the model, high fit residuals, local dependency, or DIF. Unidimensionality and overall model fit was assessed once issues with items breaching the above assumptions had been resolved. Further details of the IRT analyses are given in Multimedia Appendix 1.

#### Computer Adaptive Testing

CAT is a process whereby items from an item bank are automatically chosen and administered one-by-one, based on an algorithm that attempts to choose items that will maximize the information gained about the test taker. While CATs may be of any length, they are usually governed by a “stopping rule.” Estimations of person location on the underlying continuum (their level of QoL, in this context) are recalculated depending on previous item responses, and the item that has the greatest information function (IF) at the reestimated level of theta is then administered. This estimation process continues until the stopping rule is met. Stopping rules may demand that a questionnaire is finished once a certain number of items have been administered, or the test has been going on for a predefined amount of time, or until a level of measurement precision has been achieved. Measurement precision is defined using the standard error (SE) of measurement. SE is inversely related to (and thus comparable with) marginal reliability such that reliability=1 – SE^2^, where the standard deviation of the distribution is equal to 1.

**Figure 1:**
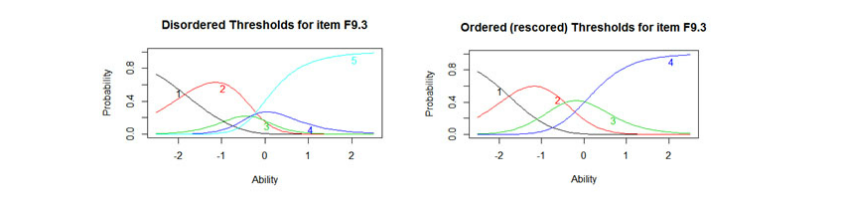
Disordered and reordered thresholds for item F9.3 “How much do any difficulties in mobility bother you?” (F9.3 has been rescored from 1-2-3-4-5 to 1-2-3-3-4 to account for the disordered category thresholds 3 and 4).

#### Simulation

We used the R-based CAT simulation engine Firestar [50] to simulate CATs in this study. The first item that the CAT administered for each domain was the item with the greatest information at the distribution mean. The IRT scaling constant was set to 1.7 [51]. We conducted 1000 iterations of the CAT with data simulated using distribution of person location (theta) values based on PCM estimations from the current dataset [47,52].

For this study, we defined three stopping rules for the CAT simulations based on standard errors equivalent to reliability values of .70, .80, and .90 (SE=.55, .45 and .32, respectively). These values were picked because they represent the minimum value for group measurement (.70) and the minimum value for individual-level measurement (.90), as well as a value in between [53]. Comparative analysis was facilitated by running a second set of simulations with stopping rules based on the published reliability values of the paper-based versions of the WHQOL-100 and the WHOQOL-BREF [6,54]. For example, where the published reliability for the Psychological QoL domain was .82, we set the stopping rule standard error to .42 (which is equivalent to alpha=.82) and compared the mean number of items administered before the stopping rule was met, with the number of items in the paper-based questionnaire.

Firestar uses a Bayesian expected a posteriori theta estimator and the maximum posterior weighted information (MPWI) item selection criterion. The MPWI selects items based on the IF weighted by the posterior distribution of trait/phenomena values [55]. This criterion has been shown to provide excellent measurement information for CAT using polytomous items.

#### Software

Analyses were conducted using Rasch Unidimensional Measurement Models 2030 [56] and the R Statistical Computing Environment [57] with the “mokken” and “ltm” packages installed [58-60]. Rasch analysis was conducted solely using RUMM, and “ltm” was used to draw Figure 1. Computer adaptive testing simulation was conducted using the FIRESTAR code generator for R [50].

## Results

The 100 items of the WHOQOL-100 were arranged into four subscales, reflecting the factor structure of the shorter WHOQOL-BREF measure [42,54].

### Domain One: Physical Quality of Life

Mokken analysis confirmed the initial scalability of all 28 items in the physical QoL subscale. All items returned a Ho value >.30 indicating acceptable scalability (see Multimedia Appendix 1 for the details of items removed from the scale).

Following Mokken analysis, the 28 items were fitted to the PCM to evaluate their advanced psychometric properties. Initial fit to the Rasch model was poor (see Table 1, Physical Initial). A number of items displayed disordered threshold and were rescored (see Figure 1 for an example of disordered and ordered thresholds). Misfit was apparently driven by 14 items that displayed high fit residuals (≥ ±1.4) and six locally dependent items, resulting in the removal of a total of 17 items. Details of the items removed from each domain may be found in Multimedia Appendix 2.

The final Physical QoL item bank consisted of 11 items, with excellent fit to the PCM and high reliability (see Table 1, analysis Physical Final). The scale was free from DIF and local dependency and was unidimensional (final item fit statistics, scoring, and threshold locations are shown in Multimedia Appendix 3). The item bank was well targeted, with fewer than 1.2% extreme scores.

### Domain Two: Psychological Quality of Life

Mokken analysis indicated the removal of six items from Psychological facets on “Thinking” (1 item), “Self-esteem” (2 items), and “Spiritual” (3 items) domain, which did not scale appropriately (Ho <.30) with the rest of the items in the scale.

Following removal of the six items, the remaining 18 items did not fit the PCM (χ^2^_216_=474.6, *P*<.001; see Table 1, analysis Psychological Initial). A number of items required rescoring to account for disordered thresholds. Misfit was driven by three items with high positive fit residuals and three items displaying local dependency (see Multimedia Appendix 1). Following removal of these items, the final scale showed excellent fit to the Rasch model, including excellent reliability, targeting, unidimensionality, and an absence of DIF or local dependency (see Table 1, Psychological Final).

### Domain Three: Social Quality of Life

Mokken analysis confirmed the scalability of the 12 items (Ho >.30; see Multimedia Appendix 1) but these 12 items did not fit the PCM (χ^2^_106_=143.49, *P*<.001; see Table 1, analysis Social Initial). A number of items were rescored to resolve issues caused by disordered thresholds. Misfit was driven by four locally dependent items (14.2, 15.1, 14.3, and 15.2; see Multimedia Appendix 1). Following removal of these items, the final 8-item scale fit the Rasch model (χ^2^_72_=88.37, *P*=.09; see Table 1, analysis Social Final) including the absence of DIF or local dependency and excellent reliability, and unidimensionality. The Social QoL item bank was also exceptionally well targeted, with only 3 respondents falling outside the measurable range of the scale (0.94%).

### Domain Four: Environmental Quality of Life

Mokken analysis indicated the removal of 16 items from the 32-item Environmental QoL scale (Loevinger’s Ho <0.3; see Multimedia Appendix 1). The remaining 16 items did not fit the PCM (χ^2^_144_=191.23, *P*<.001). The iterative item removal and rescoring procedure led to a reduction of seven items that breached the assumption of local dependency (see Multimedia Appendix 1). The final scale has an excellent fit to the Rasch model (χ^2^_81_=65.11, *P*=.90) including good reliability, excellent scale targeting, and acceptable dimensionality (see Table 1, Environmental Final).

Table 1 displays overall summaries for the initial and final analyses performed to validate each item bank. None of the item banks showed immediate fit to the Rasch model without modification.

**Table 1:** Summary Rasch fit statistics and psychometric criteria for all subscales.

Analysis ID^a^		Items n	Item residual		Person residual		Chi square			Reliability	Extremes, %	% of *t* tests significant	*t* test 95% CI
			Mean	SD	Mean	SD	χ^2^	df	*P*				
Physical	Initial	28	.53	2.7	-.28	1.71	388.5	171	<.01	.91	0	30	15.62-24.38
	Final	11	.32	1.2	-.37	1.42	109	108	0.46	.89	1.56	4.5	2.13-6.61
Psychological	Initial	24	.67	2.6	-.29	1.91	474.6	216	<.01	.91	0	22.85	18.21-27.49
	Final	14	.31	1.3	-.35	1.52	133.6	135	.52	.90	0	7.18	4.35-10.01
Social	Initial	12	.39	2	-.34	1.36	143.5	106	<.01	.87	0	17.58	13.36-21.8
	Final	8	-.03	1.5	-.38	1.05	88.37	72	.09	.81	.94	8.78	5.67-11.89
Environmental	Initial	16	.30	1.3	-.39	1.54	191.2	144	<.01	.88	0	8.86	5.73-11.99
	Final	9	.38	0.9	-.32	1.18	65.11	81	.90	.80	0	7.5	4.61-10.39
Ideal values			0	>1.4	0	>1.4			>0.01	>0.85	< 10%	<5%	<5%

^a^“Initial” refers to preanalysis values, “Final” to the final version.

**Table 2:** Summary of computer adaptive testing (CAT) simulation (1000 iterations).

Domain QoL	Stopping rule, SE(θ)	Number of items used		Range of items used	Mean SE	Reliability	Correlation between CAT θ and complete test θ
		Mean	SD				
Physical	<.32	10.01	1.22	8-11	0.32	0.9	1
	<.45	4.23	0.84	3-6	0.43	0.82	0.99
	<.55	2.46	0.5	2-3	0.52	0.73	0.98
Psychological	<.32	9.8	2	7-12	0.32	0.9	1
	<.45	4.5	0.94	3-6	0.42	0.82	0.98
	<.55	4.32	0.45	4-6	0.52	0.73	0.96
Social	<.32	7.3	1.06	5-8	0.36	0.87	1
	<.45	4.32	1.71	3-8	0.42	0.82	0.99
	<.55	2.44	0.7	2-4	0.5	0.75	0.97
Environmental	<.32	7.96	1.25	6-9	0.34	0.89	1
	<.45	3.61	1.39	2-7	0.43	0.82	0.98
	<.55	2.34	0.48	2-4	0.48	0.77	0.97

**Table 3:** Comparison of paper-based World Health Organization Quality of Life (WHOQOL) measures and the computer adaptive testing (CAT) simulations of the item banks.

Scale	Domain	Original scale information		Stopping rule	CAT simulation	
		Items, n	Reliability, alpha	Reliability-matched SE	Items administered, median	Actual SE
WHOQOL-BREF	Physical	7	0.82	0.42	4	0.42
	Psychological	6	0.81	0.44	4	0.42
	Social	3	0.68	0.55	2	0.5
	Environmental	8	0.8	0.45	3	0.43
WHOQOL-100^a^	Physical	16	0.86	0.37	7	0.36
	Psychological	20	0.82	0.42	4	0.42
	Social	12	0.73	0.52	2	0.5
	Environmental	32	0.85	0.39	5	0.38

^a^Independence and spirituality domains omitted.

### Computer Adaptive Testing Simulations

The results of the initial computer adaptive testing (CAT) simulation are displayed in Table 2. Predefined stopping rules based on different SE values were used to assess the number of items that the CAT needed to administer to reach a given level of reliability. Despite the relatively small item banks, acceptable reliability was gained with a mean of four items across all administrations (alpha>.70) and a high standard of reliability (alpha>.90) could be gained with a mean of 9 items (alpha>.90).

The results of the CAT simulation for each item bank are presented in Table 3. Stopping rules based on SE values yielded tests of varying lengths. The reduced item versions correlated strongly with the full-length item banks.

A second reliability-matched simulation (where the stopping rule of the simulation was matched to the reliability of the published measures [6,54]) shows that the item banks can produce a measurement that is as reliable as the WHOQOL-BREF and the WHOQOL-100 using 43% and 75% fewer items, respectively.

## Discussion

### Principal Findings

We calibrated four item banks that measure physical, psychological, social, and environmental QoL. Simulated computer-adaptive administration of the item banks demonstrates their ability to create accurate measurements that are both significantly shorter and often more reliable than paper-based alternatives.

In this study, the decision was made to evaluate an item bank on a sample collected in the United Kingdom. This sample was chosen to avoid issues of differential item functioning that has had a significant impact on previous studies using multinational data [32], and with the aim of providing an item bank that could be used to inform a CAT suitable for use within clinical practice.

While this study plainly demonstrates the advantages of IRT and CATs in terms of their reliability and efficiency, these techniques can also improve the quality of clinical PROMs by other means. The ability of the algorithms to select the most relevant items based on a patient’s previous responses may also provide utility in clinical measurement. Targeting in this manner not only makes assessments more relevant but also prevents patients from being asked to complete items that may be distressing or redundant. For example, a person at an early stage of a progressive disease may become distressed or concerned by completing items that assess symptoms they may experience in many later stages of the disease. A correctly calibrated item bank and CAT administration system could create accurate measurement for such patients without the need to present items that were not relevant to their level of functional impairment.

The results of this study are in line with findings from prior investigations of item bank performance, most notably and recently from the PROMIS group, insofar as CAT produced measurement estimates that were more precise, efficient, and flexible than paper-based tests for other constructs including fatigue and functional status (eg, [61-63]). To our knowledge, this study represents the first time that simulations of generic QoL item banks have been tested in this manner, though a recent study has developed an item bank suitable for assessing emotional functioning in cancer [64].

Previous studies that have applied WHOQOL scale data IRT models have employed different approaches. Studies using WHOQOL-100 data and the Rasch model have evaluated the suitability of an “index” solution, which assesses QoL as a single unidimensional, rather than multidimensional, construct. In these studies, the strict assumptions of the Rasch model led to the removal of a similar number of items, though they were not the same items that we removed from this study. Issues of DIF were also evident [32]. Other IRT analyses reported elsewhere have often presented caveats such as poor reliability or unclear dimensionality for one or more of the subscales, especially on analysis using the shorter WHOQOL-BREF (eg, [33,45]).

One notable advantage of the methods employed in the current analysis of the larger initial item banks (eg, using the WHOQOL-100 items arranged into the WHOQOL-BREF format) led to acceptable measurement across all four domains of the WHOQOL measure and obtaining excellent measurement properties with each. It must be noted that the Social QoL domain displayed unidimensionality that was slightly above the recommended threshold (5.67%, rather than 5%) for strict unidimensionality.

Multi-item questionnaires measuring health outcomes are still widely used in clinical trials [65] and epidemiological research [66]. Due to the wide variance in the type and function of PROMs, it is no small task to develop recommendations for how often they should be recalibrated using contemporary data. Happily, the increased use of IRT and adaptive testing, rather than classical test theory, means that is possible to engage in a process of iterative calibration, and the addition of new items to an item bank while collecting data for other purposes. This practice of pretesting is common in educational testing, where items must be frequently changed to reduce cheating [67,68].

The cross-cultural development of the original WHOQOL instruments suggests good potential for the development of culturally sensitive item banks and CATs. Further analyses to the one presented here provided preliminary evidence on the use of the WHOQOL item banks for use in different cultures (eg, [69,70]).

From a technical perspective, there is clear potential to develop the IRT methods employed in this study further and to apply these data to a multidimensional item response theory (MIRT) model [71]. An MIRT solution using the bi-factor model could take account of the shared variance between items in the four domains to simultaneously produce a summary score for global QOL alongside the scores for the individual domains [49]. Sample size restrictions precluded such an analysis being conducted in our study. We must note that while such a bi-factor MIRT analysis would be cutting edge regarding its methodology, some work is yet to be done to demonstrate the clinical relevance and interpretability of MIRT questionnaires and adaptive tests, though multidimensional computer adaptive tests are beginning to emerge [72].

This study naturally provides the foundations for future work to develop and evaluate a CAT system than can deliver these item banks to clinical populations and to assess the performance of the item banks under “live” testing conditions, rather than use simulations. The recent development of free-to-use and open source CAT platforms, such as Concerto [29] opens the possibility for the widespread use of computer-assisted psychometrics in clinical research and practice. Additionally, the adoption of the WHOQOL questionnaire offers the availability of 15 different language-translated versions of the questionnaire items, increasing the feasibility of international assessment of QoL using CATs [29,70].

### Conclusion

We have presented functional item banks that are capable of producing high-quality measurement across four domains of QoL using fewer items than equivalent paper-based measures. These item banks outperform the paper-based versions of the WHOQOL both in terms of reliability, length, and the flexibility in which they may be administered. The computer adaptive tests based on the WHOQOL would be suitable across a range of medical specialities, and hence particularly useful in primary care as an aid to understanding and quantifying the quality of life across diverse biopsychosocial domains.

## Appendix

Multimedia Appendix 1 Additional details of item response theory analysis procedure.

Multimedia Appendix 2 Details of removed items.

Multimedia Appendix 3 Summary item fit statistics.

## Abbreviations

CAT: computer adaptive testing
DIF: differential item functioning
IRT: item response theory
MPWI: maximum posterior weighted information
PCC: patient-centered care
PROMIS: patient-reported outcomes assessment system
PROMS: patient-reported outcome measures
QoL: quality of life
SE: standard error
WHO: World Health Organization
WHOQOL-100: World Health Organization Quality of Life 100 questionnaire
WHOQOL BREF: World Health Organization Brief questionnaire
MIRT: multidimensional item response theory

## References

[1] GBD 2013 Mortality and Causes of Death Collaborators (2015). Global, regional, and national age–sex specific all-cause and cause-specific mortality for 240 causes of death, 1990–2013: a systematic analysis for the Global Burden of Disease Study 2013. The Lancet.

[2] Committee on Quality of Health Care in America Institute of Medicine (2001). Crossing the Quality Chasm?: A New Health System for the 21st Century.

[3] Stewart M (2001). Towards a global definition of patient centred care. BMJ.

[4] Roter D (2000). The enduring and evolving nature of the patient–physician relationship. Patient Education and Counseling.

[5] Department of Health (2012). The NHS Outcomes Framework 2013/14.

[6] Skevington S, Lotfy M, O'Connell K (2004). The World Health Organization's WHOQOL-BREF quality of life assessment: Psychometric properties and results of the international field trial. A Report from the WHOQOL Group. Qual Life Res.

[7] Campbell J, Smith P, Nissen S, Bower P, Elliott M, Roland M (2009). The GP Patient Survey for use in primary care in the National Health Service in the UK--development and psychometric characteristics. BMC Fam Pract.

[8] Hibbard JH, Stockard J, Mahoney ER, Tusler M (2004). Development of the Patient Activation Measure (PAM): conceptualizing and measuring activation in patients and consumers. Health Serv Res.

[9] Skevington SM (1999). Measuring quality of life in Britain. Journal of Psychosomatic Research.

[10] US Food and Drug Administration (2006). Health and Quality of Life Outcomes.

[11] Valderas JM, Kotzeva A, Espallargues M, Guyatt G, Ferrans CE, Halyard MY, Revicki DA, Symonds T, Parada A, Alonso J (2008). The impact of measuring patient-reported outcomes in clinical practice: a systematic review of the literature. Qual Life Res.

[12] Skevington SM, Day R, Chisholm A, Trueman P (2005). How much do doctors use quality of life information in primary care? Testing the Trans-Theoretical Model of behaviour change. Qual Life Res.

[13] Marshall S, Haywood K, Fitzpatrick R (2006). Impact of patient-reported outcome measures on routine practice: a structured review. J Eval Clin Pract.

[14] Greenhalgh J, Meadows K (1999). The effectiveness of the use of patient-based measures of health in routine practice in improving the process and outcomes of patient care: a literature review. J Eval Clin Pract.

[15] Velikova G, Keding A, Harley C, Cocks K, Booth L, Smith A, Wright P, Selby PJ, Brown JM (2010). Patients report improvements in continuity of care when quality of life assessments are used routinely in oncology practice: secondary outcomes of a randomised controlled trial. Eur J Cancer.

[16] Aaronson N (1999). Assessing quality of life in clinical practice in oncology. Eur J Cancer.

[17] Wolfe J, Orellana L, Cook E, Ullrich C, Kang T, Geyer J (2014). Improving the care of children with advanced cancer by using an electronic patient-reported feedback intervention: results from the PediQUEST randomized controlled trial. J Clin Oncol.

[18] Velikova G, Booth L, Smith A, Brown P, Lynch P, Brown J, Selby PJ (2004). Measuring quality of life in routine oncology practice improves communication and patient well-being: a randomized controlled trial. J Clin Oncol.

[19] DuBenske L, Gustafson D, Namkoong K, Hawkins R, Brown R, McTavish F (2010). Effects of an interactive cancer communication system on lung cancer caregivers' quality of life and negative mood: a randomized clinical trial.

[20] Goncalves DCB, Gibbons C, Ricci-Cabello I, Bobrovitz N, Gibbons E, Kotzeva A, Alonso J, Fitzpatrick R, Bower P, van der Wees P, Rajmil L, Roberts N, Taylor R, Greenhalgh J, Porter I, Valderas J (2015). Routine provision of information on patient-reported outcome measures to healthcare providers and patients in clinical practice. Cochrane Database Syst Rev.

[21] Llewellyn A, Skevington S (2015). Using guided individualised feedback to review self-reported quality of life in health and its importance. Psychol Health.

[22] Llewellyn AM, Skevington SM (2016). Evaluating a new methodology for providing individualized feedback in healthcare on quality of life and its importance, using the WHOQOL-BREF in a community population. Qual Life Res.

[23] Krägeloh CU, Czuba KJ, Billington DR, Kersten P, Siegert RJ (2015). Using feedback from patient-reported outcome measures in mental health services: a scoping study and typology. Psychiatr Serv.

[24] Jani BD, Purves D, Barry S, Cavanagh J, McLean G, Mair FS (2013). Challenges and implications of routine depression screening for depression in chronic disease and multimorbidity: a cross sectional study. PLoS One.

[25] Dawson J, Doll H, Fitzpatrick R, Jenkinson C, Carr AJ (2010). The routine use of patient reported outcome measures in healthcare settings. BMJ.

[26] Nakash RA, Hutton JL, Jørstad-Stein EC, Gates S, Lamb SE (2006). Maximising response to postal questionnaires--a systematic review of randomised trials in health research. BMC Med Res Methodol.

[27] Jette AM, McDonough CM, Haley SM, Ni P, Olarsch S, Latham N, Hambleton RK, Felson D, Kim Y, Hunter D (2009). A computer-adaptive disability instrument for lower extremity osteoarthritis research demonstrated promising breadth, precision, and reliability. J Clin Epidemiol.

[28] Petersen MA, Aaronson NK, Arraras JI, Chie W, Conroy T, Costantini A, Giesinger JM, Holzner B, King MT, Singer S, Velikova G, Verdonck-de Leeuw IM, Young T, Groenvold M, EORTC Quality of Life Group (2013). The EORTC computer-adaptive tests measuring physical functioning and fatigue exhibited high levels of measurement precision and efficiency. J Clin Epidemiol.

[29] Psychometrics Centre (2013). Concerto Adaptive Testing Platform.

[30] Scalise K, Allen DD (2015). Use of open-source software for adaptive measurement: Concerto as an R-based computer adaptive development and delivery platform. Br J Math Stat Psychol.

[31] Amtmann D, Cook KF, Jensen MP, Chen W, Choi S, Revicki D, Cella D, Rothrock N, Keefe F, Callahan L, Lai J (2010). Development of a PROMIS item bank to measure pain interference. Pain.

[32] Leplege A, Ecosse E, WHOQOL Rasch Project Scientific Committee (2000). Methodological issues in using the Rasch model to select cross culturally equivalent items in order to develop a Quality of Life index: the analysis of four WHOQOL-100 data sets (Argentina, France, Hong Kong, United Kingdom). J Appl Meas.

[33] Pomeroy IM, Tennant A, Young CA (2013). Rasch analysis of the WHOQOL-BREF in post polio syndrome. J Rehabil Med.

[34] Lai J, Cella D, Chang C, Bode R, Heinemann A (2003). Item banking to improve, shorten and computerize self-reported fatigue: an illustration of steps to create a core item bank from the FACIT-Fatigue Scale. Qual Life Res.

[35] Forkmann T, Boecker M, Norra C, Eberle N, Kircher T, Schauerte P, Mischke K, Westhofen M, Gauggel S, Wirtz M (2009). Development of an item bank for the assessment of depression in persons with mental illnesses and physical diseases using Rasch analysis. Rehabil Psychol.

[36] Gibbons CJ, Mills RJ, Thornton EW, Ealing J, Mitchell JD, Shaw PJ, Talbot K, Tennant A, Young CA (2011). Rasch analysis of the hospital anxiety and depression scale (HADS) for use in motor neurone disease. Health Qual Life Outcomes.

[37] Bee P, Gibbons C, Callaghan P, Fraser C, Lovell K (2016). Evaluating and Quantifying User and Carer Involvement in Mental Health Care Planning (EQUIP): Co-Development of a New Patient-Reported Outcome Measure. PLoS ONE.

[38] Perline R, Wright BD, Wainer H (1979). The Rasch Model as Additive Conjoint Measurement. Applied Psychological Measurement.

[39] Andrich D (2004). Controversy and the Rasch model: a characteristic of incompatible paradigms?. Med Care.

[40] Karabatsos G (2001). The Rasch model, additive conjoint measurement, and new models of probabilistic measurement theory. J Appl Meas.

[41] Smits N, Cuijpers P, van Straten A (2011). Applying computerized adaptive testing to the CES-D scale: a simulation study. Psychiatry Res.

[42] The WHOQOL Group (1998). Development of the World Health Organization WHOQOL-BREF quality of life assessment. The WHOQOL Group. Psychol Med.

[43] The WHOQOL Group (1995). The World Health Organization quality of life assessment (WHOQOL): Position paper from the World Health Organization. Social Science & Medicine.

[44] Skevington SM, McCrate FM (2012). Expecting a good quality of life in health: assessing people with diverse diseases and conditions using the WHOQOL-BREF. Health Expect.

[45] Rocha NS, Power MJ, Bushnell DM, Fleck MP (2012). Cross-cultural evaluation of the WHOQOL-BREF domains in primary care depressed patients using Rasch analysis. Med Decis Making.

[46] Schmidt S, Mühlan H, Power M (2006). The EUROHIS-QOL 8-item index: psychometric results of a cross-cultural field study. Eur J Public Health.

[47] Masters GN (1982). A Rasch model for partial credit scoring. Psychometrika.

[48] Engelhard G (2008). Historical Perspectives on Invariant Measurement: Guttman, Rasch, and Mokken. Measurement: Interdisciplinary Research and Perspectives.

[49] Gibbons CJ, Kenning C, Coventry PA, Bee P, Bundy C, Fisher L, Bower P (2013). Development of a multimorbidity illness perceptions scale (MULTIPleS). PLoS One.

[50] Choi S (2009). Firestar: Computerized Adaptive Testing Simulation Program for Polytomous Item Response Theory Models. Applied Psychological Measurement.

[51] Camilli G (1994). Origin of the scaling constant d=1.7 in Item Response Theory. J Educ Behav Stat.

[52] Choi SW, Reise SP, Pilkonis PA, Hays RD, Cella D (2010). Efficiency of static and computer adaptive short forms compared to full-length measures of depressive symptoms. Qual Life Res.

[53] Reeve B, Hays R, Bjorner J, Cook K, Crane P, Teresi J, Thissen D, Revicki DA, Weiss DJ, Hambleton RK, Liu H, Gershon R, Reise SP, Lai J, Cella D (2007). Psychometric evaluation and calibration of health-related quality of life item banks: plans for the Patient-Reported Outcomes Measurement Information System (PROMIS). Med Care.

[54] The WHOQOL Group (1998). The World Health Organization Quality of Life Assessment (WHOQOL): development and general psychometric properties. Soc Sci Med.

[55] Choi SW, Swartz RJ (2009). Comparison of CAT Item Selection Criteria for Polytomous Items. Appl Psychol Meas.

[56] Andrich D, Sheridan B, Luo G (2010). Rasch models for measurement: RUMM2030.

[57] R Development Team R: A language and environment for statistical computing.

[58] van der Ark LA (2007). Mokken Scale Analysis in R. J Stat Soft.

[59] van der Ark LA (2007). New Developments in Mokken Scale Analysis in R. J Stat Soft.

[60] Rizopoulos D (2006). ltm: An R Package for Latent Variable Modeling and Item Response Theory Analyses. J Stat Soft.

[61] Cella D, Gershon R, Lai J, Choi S (2007). The future of outcomes measurement: item banking, tailored short-forms, and computerized adaptive assessment. Qual Life Res.

[62] Lai J, Cella D, Chang C, Bode R, Heinemann A (2003). Item banking to improve, shorten and computerize self-reported fatigue: An illustration of steps to create a core item bank from the FACIT-Fatigue Scale. Qual Life Res.

[63] Fries JF, Cella D, Rose M, Krishnan E, Bruce B (2009). Progress in assessing physical function in arthritis: PROMIS short forms and computerized adaptive testing. J Rheumatol.

[64] Petersen MA, Gamper E, Costantini A, Giesinger JM, Holzner B, Johnson C, Sztankay M, Young T, Groenvold M, EORTC Quality of Life Group (2016). An emotional functioning item bank of 24 items for computerized adaptive testing (CAT) was established. J Clin Epidemiol.

[65] Pi-Sunyer X, Astrup A, Fujioka K, Greenway F, Halpern A, Krempf M, Lau D, le RC, Violante OR, Jensen CW (2015). A randomized, controlled trial of 30 mg of liraglutide in weight management. New Engl J Med.

[66] Eekhout I, Enders CK, Twisk JWR, de Boer MR, de Vet HCW, Heymans MW (2015). Including auxiliary item information in longitudinal data analyses improved handling missing questionnaire outcome data. J Clin Epidemiol.

[67] Crocker L, Algina J (1986). Introduction to Classical and Modern Test Theory.

[68] Parshall C (2002). Item Development and Pretesting in a CBT Environment. Building the foundations for future assessments.

[69] Tennant A, Penta M, Tesio L, Grimby G, Thonnard J, Slade A, Lawton G, Simone A, Carter J, Lundgren-Nilsson A, Tripolski M, Ring H, Biering-Sørensen F, Marincek C, Burger H, Phillips S (2004). Assessing and adjusting for cross-cultural validity of impairment and activity limitation scales through differential item functioning within the framework of the Rasch model: the PRO-ESOR project. Med Care.

[70] Gibbons C (2015). Assessing the performance of an item bank derived from the World Health Organisation Quality of Life-100 measure for computer adaptive testing across diverse cultures. Qual Life Res.

[71] Chalmers R (2012). mirt: A Multidimensional Item Response Theory Package for the Environment. J Stat Soft.

[72] Hackshaw M (2010). Association of patient-reported outcomes with progression-free survival in malignant pleural mesothelioma. Diss Abstr Int Sect B Sci Eng.

