# Supplementary material for: Electronic Quality of Life Assessment Using Computer-Adaptive Testing
Source: J Med Internet Res. 2016 Sep 30;18(9):e240. doi: 10.2196/jmir.6053 (PMC5065679; doi:10.2196/jmir.6053)
Supplement: Multimedia Appendix 1 [file jmir_v18i9e240_app1.pdf]

Appendix A – Details of item response theory analysis

Mokken analysis

Mokken analysis was employed in this study to ensure that all items analysed using Rasch analysis were sufficiently scalable. Scalability is identified using Loevinger's H value. Mokken (1971) suggested several 'rules of thumb' for assessing Loevinger's scalability coefficients, A scale is considered weak if $.3 \leq H <$ $.4$, considered 'moderately scalable' if $.4 \leq H < .5$ and strong if $H \geq .5$.

Mokken analysis is employed prior to Rasch analysis to ensure that the banks contain probabilistic structure that would be consistent with the Rasch model [1]. A Loevinger's coefficient value of < 0.3 would indicate an item not consistent with this structure [2]. Mokken is better suited to analysing unidimensional scales with bi-polar conceptual structure than factor analysis, as factor analysis sometimes erroneously splits bi-polar concepts into different factors [3,4]. Mokken analysis is a recommended technique to use prior to parametric IRT analyses, including Rasch analysis, as it can reduce error in subsequent analyses [5] and indicate dimensionality, in a similar fashion to factor analysis [6,7], but without some of the issues of this approach[8].

Rasch analysis

The Rasch model is closely related to parametric item response theory models. It is considered to be the 'practical realisation' ([9] p.237) of Luce and Tukey's [10] additive conjoint measurement, allowing the social sciences, which must deal with latent traits and other phenomena that are not directly observable, to confirm the construction of fundamental measurement for latent phenomena[11]. Therefore, when scale data are successfully fitted to the Rasch model they can be said to capable of linear unidimensional measurement. We selected the Rasch model as it is sample-distribution independent, so maintaining specific objectivity (allowing comparisons to be made between individuals independent of the instruments which are used)[45]. In our experience, fitting scale data to the Rasch model has created efficient (few items with greater reliability) and precise (reduced measurement error) paper-based questionnaire measures in diverse areas of the health sciences[46–49]. The Rasch model has previously been used to successfully develop item banks for fatigue [50], depression [51] and pain [52].

Rasch analysis follows an iterative process of scale evaluation, modification and re-assessment. The cardinal criterion is scale fit to the Rasch model, indicated by a non-significant chi square interaction between the model and the data (Chi Square probability > 0.01). If the scale data does not fit the Rasch model it is necessary to establish the reasons for the misfit. Indicators that may identify the reasons for misfit include category threshold ordering, item fit residuals, differential item functioning, local dependence and dimensionality. A brief explanation of each indicator is given below; further information is available elsewhere[12].

Category Threshold Analysis

Scales with polytomous response modalities have several ordered response categories (*e.g.* a Likert scale) which are typically scored sequentially with a higher score indicating a higher level of the latent phenomena being measured. Categories can become disordered when the category is not modal, i.e., respondents to not endorse it frequently enough[13]. Categories that are disordered may be collapsed adjacently and rescored in order to preserve the correct ordering. Disordered category thresholds prevent the calculation of interval level estimates from the item banks and have a negative impact on overall model fit.

Item fit residual

Item fit residuals are analysed to ascertain if the individual items fit the Rasch model or if the items are over (high negative fit residual) or under (high positive fit residual) discriminating. Items that under-discriminate are considered to have a weak relationship with the underlying construct and those with a high fit residual are likely to correlate too strongly with the underlying construct, indicating possible redundancy or dependency with other items. For the current analysis, items with a fit residual greater than ±2.5 logits were removed from the scale.

Differential item functioning

Differential item functioning (DIF) occurs when different demographic groups in a sample respond in a systematically different way to an item. Two types of DIF can occur; uniform DIF, where a certain group responds differently across the entire range of the underling phenomena; and non-uniform DIF, where a group responds differently to an item at a certain level of the underlying phenomena. In the current study we analysed DIF by gender, age group and broad medical status (well/sick) using ANOVA.

Local dependency

Local dependency is assessed statistically by correlating item residuals, correlations greater than +. 2 are considered to be locally dependent[12] and the item should either be removed from the scale or 'collapsed' into a testlet (a bundle of common items)[14]. The best strategy for dealing with local dependency (deletion or testlet) is determined by the capacity of future test administrators (whether they be computers or humans) to take account of locally dependent items in their administration protocol. As many CAT simulation and administration programs do not yet have the functionality to account for local dependency in this manner, we decided to remove locally dependent items, rather than collapse into a testlet. For the current analysis, where pairs of items are locally dependent, the item with the greatest (positive or negative) fit residual will be removed.

Dimensionality

A fundamental assumption of item banks for clinical purposes is that the items within each bank all measure the same single underlying phenomenon (*e.g.* psychological QoL). This is known as the assumption of unidimensionality and it is assessed using a formal test of the difference between component loadings on the first residual factor within the scale[15]. A principal components analysis is conducted and items are divided into two groups: those that load positively and those that load negatively on the first residual factor. Both sets of items are then used to create an independent estimate for each participant. An independent samples *t*-test is then conducted to assess if there is a significant difference between the two estimates for all of the participants in the sample. As the scale is expected to by unidimensional, the hypothesis is that there be minimal difference between the two groups. The acceptable criterion for unidimensionality is that fewer than 5% of the *t* tests return a significant result (or the 95% confidence interval falls below 5% of significant tests)[16].

References

1. Mokken RJ. *A Theory and Procedure of Scale Analysis: With Applications in Political Research.* Walter de Gruyter; 1971. https://books.google.com/books?hl=en&lr=&id=vAumIrkzYj8C&pgis=1. Accessed February 16, 2015.

2. Loevinger J. A Systematic Approach to the Construction and Evaluation of Tests of Ability. *Psychol Monogr Gen Appl.* 1947;61(4):i - 49.

3. Van Schuur WH. Mokken Scale Analysis: Between the Guttman Scale and Parametric Item Response Theory. *Polit Anal.* 2003;11(2):139-163. doi:10.1093/pan/mpg002.

4. Schuur W van. *Ordinal Item Response Theory: Mokken Scale Analysis.*; 2011. https://books.google.co.uk/books?hl=en&lr=&id=8Igk82X--xIC&oi=fnd&pg=PR1&dq=Ordinal+Item+Response+Theory:+Mokken+Scale+Analysis&ots=7T4ImyLkfU&sig=K8ZYlzdb_HnBhAKSEqGdaoJk244. Accessed February 19, 2015.

5. Meijer RR, Baneke JJ. Analyzing Psychopathology Items: A Case for Nonparametric Item Response Theory Modeling.

6. Wismeijer AAJ, Sijtsma K, van Assen MALM, Vingerhoets AJJM. A comparative study of the dimensionality of the self-concealment scale using principal components analysis and Mokken scale analysis. *J Pers Assess.* 2008;90(4):323-334.

7. Emons WH, Sijtsma K, Pedersen SS. Dimensionality of the hospital anxiety and depression scale (HADS) in cardiac patients: comparison of Mokken scale analysis and factor analysis. *Assessment.* 2012;19(3):337-353. doi:10.1177/1073191110384951.

8. Bond T. Too many factors in Factor Analysis? *Rasch Meas Trans.* 1994;8(1):347.

9. Perline R, Wright BD, Wainer H. The Rasch Model as Additive Conjoint Measurement. *Appl Psychol Meas.* 1979;3(2):237-255. doi:10.1177/014662167900300213.

10. Luce RD, Tukey JW. Simultaneous conjoint measurement: A new type of fundamental measurement. *J Math Psychol.* 1964;1(1):1-27. doi:10.1016/0022-2496(64)90015-X.

11. Karabatsos G. The Rasch model, additive conjoint measurement, and new models of probabilistic measurement theory. *J Appl Meas.* 2001;2(4):389-423.

12. Pallant J, Tennant A. An introduction to the Rasch measurement model: an example using the Hospital Anxiety and Depression Scale (HADS). *Br J Clin Psychol.* 2007;46(1):1-18.

13. Linacre J. Sample size and item calibration stability. 1994;7:328.

14. Wainer H, Kiely GL. Item Clusters and Computerized Adaptive Testing: A Case for Testlets. *J Educ Meas.* 1987;24(3):185-201. doi:10.1111/j.1745-3984.1987.tb00274.x.

15. Wright B. Local dependency, correlations and principal components. *Rasch Meas Trans.* 1996;10:509-511.

16. Tennant A, Pallant J. Unidimensionality matters!(A tale of two Smiths?). *Rasch Meas Trans.* 2006;20(1):1048-1051.
