# Supplementary material for: Electronic Quality of Life Assessment Using Computer-Adaptive Testing
Source: J Med Internet Res. 2016 Sep 30;18(9):e240. doi: 10.2196/jmir.6053 (PMC5065679; doi:10.2196/jmir.6053)
Supplement: Multimedia Appendix 2 [file jmir_v18i9e240_app2.pdf]

Item Removed	Parent Scale	Reason
Physical QOL Scale		
1.1	Pain	Fit residual > 1.4
1.2	Pain	Fit residual > 1.4
1.3	Pain	Fit residual > 1.4
2.2	Energy	Fit residual > 1.4
2.4	Energy	Fit residual > 1.4
3.1	Sleep	Fit residual > 1.4
3.2	Sleep	Fit residual > 1.4
3.3	Sleep	Fit residual > 1.4
3.4	Sleep	Fit residual > 1.4
9.1	Mobility	Fit residual > 1.4
9.4	Mobility	Locally dependent
11.1	Medication	Fit residual > 1.4
11.2	Medication	Fit residual > 1.4
11.3	Medication	Fit residual > 1.4
11.4	Medication	Fit residual > 1.4
12.1	Work	Locally dependent
12.3	Work	Locally dependent
Psychological QOL Scale		
5.1	Thinking	Mokken Ho ≤ .30
7.1	Esteem	Mokken Ho ≤ .30
7.2	Esteem	Mokken Ho ≤ .30
7.3	Esteem	Mokken Ho ≤ .30
24.1	Spiritual	Mokken Ho ≤ .30
24.3	Spiritual	Mokken Ho ≤ .30
24.4	Spiritual	Fit residual > 1.4
7.4	Esteem	Fit residual > 1.4
24.2	Spiritual	Fit residual > 1.4
4.4	Positive feelings	Locally dependent
6.3	Psychological	Locally dependent
8.4	Body image	Locally dependent

	Social QOL Scale	
14.2	Social	Locally dependent
15.1	Social	Locally dependent
14.3	Social	Locally dependent
15.2	Social	Locally dependent
	Environmental QOL Scale	
16.2	Safety	Mokken Ho ≤ .30
17.4	Home	Mokken Ho ≤ .30
18.4	Financial	Mokken Ho ≤ .30
19.1	Services	Mokken Ho ≤ .30
19.2	Services	Mokken Ho ≤ .30
19.3	Services	Mokken Ho ≤ .30
19.4	Services	Mokken Ho ≤ .30
21.1	Leisure	Mokken Ho ≤ .30
21.3	Leisure	Mokken Ho ≤ .30
21.4	Leisure	Mokken Ho ≤ .30
22.2	Environment	Mokken Ho ≤ .30
22.3	Environment	Mokken Ho ≤ .30
22.4	Environment	Mokken Ho ≤ .30
23.1	Transport	Mokken Ho ≤ .30
23.4	Transport	Mokken Ho ≤ .30
17.1	Home	Locally dependent
18.2	Financial	Locally dependent
23.3	Transport	Locally dependent
20.3	Information	Locally dependent
17.2	Home	Locally dependent
18.1	Financial	Locally dependent
20.1	Information	Locally dependent
