# Supplementary material for: Electronic Quality of Life Assessment Using Computer-Adaptive Testing
Source: J Med Internet Res. 2016 Sep 30;18(9):e240. doi: 10.2196/jmir.6053 (PMC5065679; doi:10.2196/jmir.6053)
Supplement: Multimedia Appendix 3 [file jmir_v18i9e240_app3.pdf]

Appendix C

Table 1C – Item fit statistics for the Physical QoL domain

Physical Quality of Life					
Item	Loc. Θ	SE	FR.	ChiSq	Prob
1.4	.26	.09	1.87	10.86	.29
2.1	-.20	.08	.66	5.52	.79
2.3	.98	.08	1.12	14.80	.10
9.2	-.37	.09	-1.11	7.70	.56
9.3	-.12	.09	2.04	12.17	.20
10.1	-.48	.08	-1.87	7.73	.56
10.2	-.51	.08	.42	22.75	.01
10.3	-.32	.10	-2.02	13.55	.14
10.4	-.26	.09	-.93	9.66	.38
12.2	.29	.08	-1.47	10.97	.28
12.4	.72	.08	2.50	10.16	.34

Key: Loc Θ = Mean item location, SE = standard error, FR = fit residual, ChiSq = Chi Squared, Prob = Probability, Thresh = Threshold

Table 2C – Item fit statistics for the Psychological QoL domain

Psycholoigcal Quality of Life					
Item	Loc. Θ	SE	FR.	ChiSq	Prob
4.1	-.57	.09	-1.25	11.43	.25
4.2	.31	.08	2.33	6.96	.64
4.3	.26	.08	-.06	6.50	.69
5.2	-.22	.08	.69	8.83	.45
5.3	.32	.08	1.08	8.79	.46
5.4	-.09	.08	-.56	4.75	.86
6.1	.08	.08	-.05	17.34	.04
6.2	.08	.08	-.47	11.42	.25
6.4	.19	.08	-.70	7.79	.56
8.1	-.05	.08	-.70	8.03	.53
8.2	.26	.07	1.15	5.77	.76
8.3	-.57	.07	2.43	11.34	.25

Key: Loc Θ = Mean item location, SE = standard error, FR = fit residual, ChiSq = Chi Squared, Prob = Probability, Thresh = Threshold

Table 3C – Item fit statistics for the Social QoL domain

Social Quality of Life					
Item	Loc. ϴ	SE	FR.	ChiSq	Prob
13.1	-.11	.07	-.49	9.83	.36
13.2	-.33	.07	-.80	10.61	.30
13.3	.11	.07	-2.90	14.03	.12
13.4	.44	.11	.42	5.46	.79
14.1	.14	.08	1.32	19.97	.02
14.4	-.35	.09	-.03	10.74	.29
15.3	.68	.07	.21	5.37	.80
15.4	-.58	.09	1.99	12.38	.19

Key: Loc ϴ = Mean item location, SE = standard error, FR = fit residual, ChiSq = Chi Squared, Prob = Probability, Thresh = Threshold

Table 4C – Item fit statistics for the Environmental QoL domain

Environmental Quality of Life					
Item	Loc. ϴ	SE	FR.	ChiSq	Prob
16.1	-.55	.08	.23	7.66	.57
16.4	.07	.08	-.16	6.52	.69
17.3	-.51	.08	-.49	13.22	.15
18.3	.76	.06	1.07	2.65	.98
20.2	-.11	.08	.10	5.70	.77
20.4	.16	.08	.06	5.46	.79
21.2	.24	.07	.49	5.64	.78
22.1	.09	.09	-.33	7.51	.58
23.2	-.16	.06	2.46	10.76	.29

Key: Loc ϴ = Mean item location, SE = standard error, FR = fit residual, ChiSq = Chi Squared, Prob = Probability, Thresh = Threshold
